# A Discouraging Opinion of Women

**Published:** 1891-04

**Authors:** 


A Discouraging Opinion of Women.—To-day, it is a fact that there are not a score of medical women who are making a decent living in all New England, and these, one-half at least, are either non-graduates, or from irregular schools.—*N. E. Medical Monthly.*

